# Health taxes: balancing public health interests and individual freedom during an era of abundant misinformation

**DOI:** 10.3389/fpubh.2026.1885569

**Published:** 2026-10-23

**Authors:** Hazem Abbas

**Affiliations:** Department of International and Community Oral Health, Graduate School of Dentistry, Tohoku University, Sendai, Japan

**Keywords:** alcohol, economics, health policy, sugar sweetened beverages, tax, tobacco

## Abstract

Health taxes on tobacco, alcohol and sugar sweetened beverages (SSBs) represent important fiscal tools for improving population health, particularly in the prevention of non-communicable diseases (NCDs) such as cardiovascular diseases, diabetes, certain cancers and even oral diseases. While concerns about individual freedom of choice and consumer sovereignty are common, valid and warrant careful consideration, health taxes do not eliminate autonomy but rather seek to rebalance the environment in which consumption choices are made. The challenges of implementing health taxes might be compounded in an era of abundant misinformation due to the ease of producing and disseminating misinformation at a scale, particularly on social networking services (SNS). Misinformation can amplify the resistance to health taxes, even among policy makers, by shifting the public sentiment to oppose health taxes through distorting scientific evidence, framing health taxes as paternalistic or coercive, and undermining trust in science and public health institutions. This was observed in debates about the COVID-19 pandemic and the community water fluoridation policies in many countries, as well as in policies aimed at reducing alcohol consumption in the United Kingdom and e-cigarettes smoking in the United States. To address these challenges, transparent communication of scientific evidence while acknowledging the knowledge gaps and uncertainties, generating clearer causal scientific evidence, sustained debunking of misinformation, public engagement in policy making, gradual phased implementation of health taxes, and combining health taxes implementation with complementary measures such as providing subsidies for healthy alternatives might be useful tools to build trust, win public support for health taxes and promote informed decision-making.

## Introduction

Health taxes on tobacco, alcohol, and sugar-sweetened beverages (SSBs) are important fiscal tools in the prevention of non-communicable diseases (NCDs) which accounted for 74% of deaths globally in 2019 (1–3). These fiscal measures are designed to reduce consumption of tobacco, alcohol and SSBs to improve population health outcomes, and generate public revenue that can be reinvested in health systems (4). However, one of the common arguments against the implementation of health taxes is that they infringe upon individual freedoms and consumer sovereignty (5–8). In other words, implementing health taxes raises enduring ethical and political questions regarding the balance between collective public health interests and individual freedom of choice (6). Recently, this tension has become increasingly complex due to the ease of spread of misinformation on social networking services (SNS) which undermine the trust in science and public institutions (9, 10).

## The debate: public health interests vs. individual autonomy

A substantial body of evidence supports the effectiveness of health taxes in reducing the consumption of tobacco, alcohol, and SSBs. Considering that tobacco was leading cause of death globally among the three, tobacco taxation, in particular, is widely regarded as one of the most effective public health interventions (11). Studies across diverse settings have shown that increases in tobacco prices lead to some reductions in smoking prevalence, especially among youth and low-income populations (11). Similarly, taxes on sugar-sweetened beverages have been associated with decreased purchases (12). For example, evaluations of SSB taxes in countries such as Mexico and the United Kingdom have demonstrated measurable declines in sales, alongside reformulation efforts by manufacturers to reduce sugar content (12–15). While, alcohol taxation has also been linked to reductions in alcohol-related harms, including injuries and chronic disease burden (16).

From a public health perspective, these findings provide strong justification for the use of taxation as a regulatory tool to reduce NCDs such as cardiovascular diseases, diabetes, certain cancers and oral diseases among others as they are driven by modifiable behavioral risk factors (1, 4, 17). Health taxes aim to alter these behaviors by modifying economic incentives, thereby addressing the structural determinants of health (1, 4). In doing so, they shift the policy focus from individual responsibility alone (i.e., victim blaming) to broader environmental and economic contexts that shape health choices (18).

However, critics including food industry representatives and libertarians argue that health taxes infringe upon individual autonomy by limiting freedom of choice, and this was often labeled with strong and alluring terms such as “nanny state, state paternalism, food police and food fascism” (6, 7). In addition, some question the effectiveness of such policies, arguing that health taxes will not change consumption behaviors and consumers would rather switch to cheaper equally unhealthy alternatives, while marking these policies as “discriminatory” and “unconstitutional” because they target specific groups of products (6, 19). From a libertarian standpoint, individuals should retain the right to make personal consumption decisions, even if those decisions entail health risks (6, 7). Health taxes, in this view, may be perceived as paternalistic interventions that diminish autonomy and coerce individuals into healthier behaviors (7). This concern is particularly prominent when policies disproportionately affect lower-income populations who tend to allocate a larger proportion of their income to the consumption of tobbaco, alcohol and SSBs (11, 13, 16, 20). Consequently, health taxes are sometimes criticized as regressive, raising questions about equity and fairness (6, 7).

On the other hand, the ethical justification for health taxes often rests on the concept of “soft paternalism” or “libertarian paternalism,” which seeks to nudge individuals toward healthier choices without eliminating freedom of choice entirely (21). In other words, health taxes do not ban products; rather, they alter the relative prices of goods, allowing individuals to continue consumption if they so choose. In this sense, they preserve autonomy while promoting healthy consumption behaviors (6, 19). Moreover, when revenues generated from health taxes are earmarked for health promotion, health services coverage, or subsidization of healthier alternatives, they can contribute to reducing health inequalities (4). For example, in Europe, SSB tax revenues in France and Portugal were used to support social security and national health services (13, 22), while in Asia, the Philippines' revenues from the “Sin Tax”, an excise tax on (tobacco, alcohol, and SSBs among other goods), were earmarked to increase health spending (23, 24). Another important consideration is that while health taxes can be regressive in a purely economic sense, their health benefits may be progressive, as lower-income populations often experience a greater burden of NCDs and may respond more strongly to price changes (6, 11, 13, 16). Furthermore, supporters of health taxes argue that the costs of NCDs are borne by society as a whole through public health systems in countries adopting universal health coverage (UNC) (11, 13, 16). In addition, health advocates argue that food and beverage industry's marketing, combined with addictive qualities of ingredients like nicotine, alcohol and sugar undermine true consumer choice, and thus justifying intervention as a correction to market failures (6, 19, 20). At its core, the debate centers on whether the governments' interest in public health and reducing healthcare costs outweighs individuals' autonomy to choose their diet.

## The effect of abundant misinformation on the debate

Winning this debate, and overcoming the challenges of shifting the public sentiment toward supporting health taxes might be more complicated during the current era of widespread misinformation, considering the ease of access to tools —particularly social media platforms—that can disseminate misinformation on a scale, and had significantly influenced public perceptions of health policies in the past (9, 10). Opposition to COVID-19 lockdowns and COVID-19 vaccines as well as community water fluoridation are two clear health policy related cases of how misinformation can distort scientific evidence, undermine trust in public health institutions, and amplify resistance to public health interventions such as health taxes even among policy makers themselves (25–27).

Furthermore and apart from these two examples, industry-funded campaigns have historically played a role in shaping narratives around tobacco, alcohol and sugar consumption, often emphasizing individual responsibility while downplaying structural determinants of health (11, 13). In the current digital age, similar opposing narratives to the health taxes can spread rapidly, complicating efforts to implement them (Figure 1). For example, industry stakeholders and others can mobilize the public using advertising campaigns on social media networks through proxy accounts and/or pay internet personalities and online influencers to build public distrust in scientific evidence and grow controversy about health taxes. This was observed in the United States during regional governments' E-cigarette prevention campaigns on X (formerly Twitter) that aimed to communicate evidence about e-cigarettes ahead of regional votes on regulations (28). Coordinated social-media activity from certain accounts and bots hijacked the prevention campaigns' hashtags and created new counter-hashtags in a manner that exhibited characteristics consistent with astroturfing (the deceptive practice of hiding the true corporate or political sponsors behind an organized campaign to make it look like a spontaneous, genuine “grassroots” movement), a practice that aim to manufacture scientific uncertainty, amplify minority or unsupported interpretations of evidence, and create a false perception of scientific controversy (28, 29).

**Figure 1:**
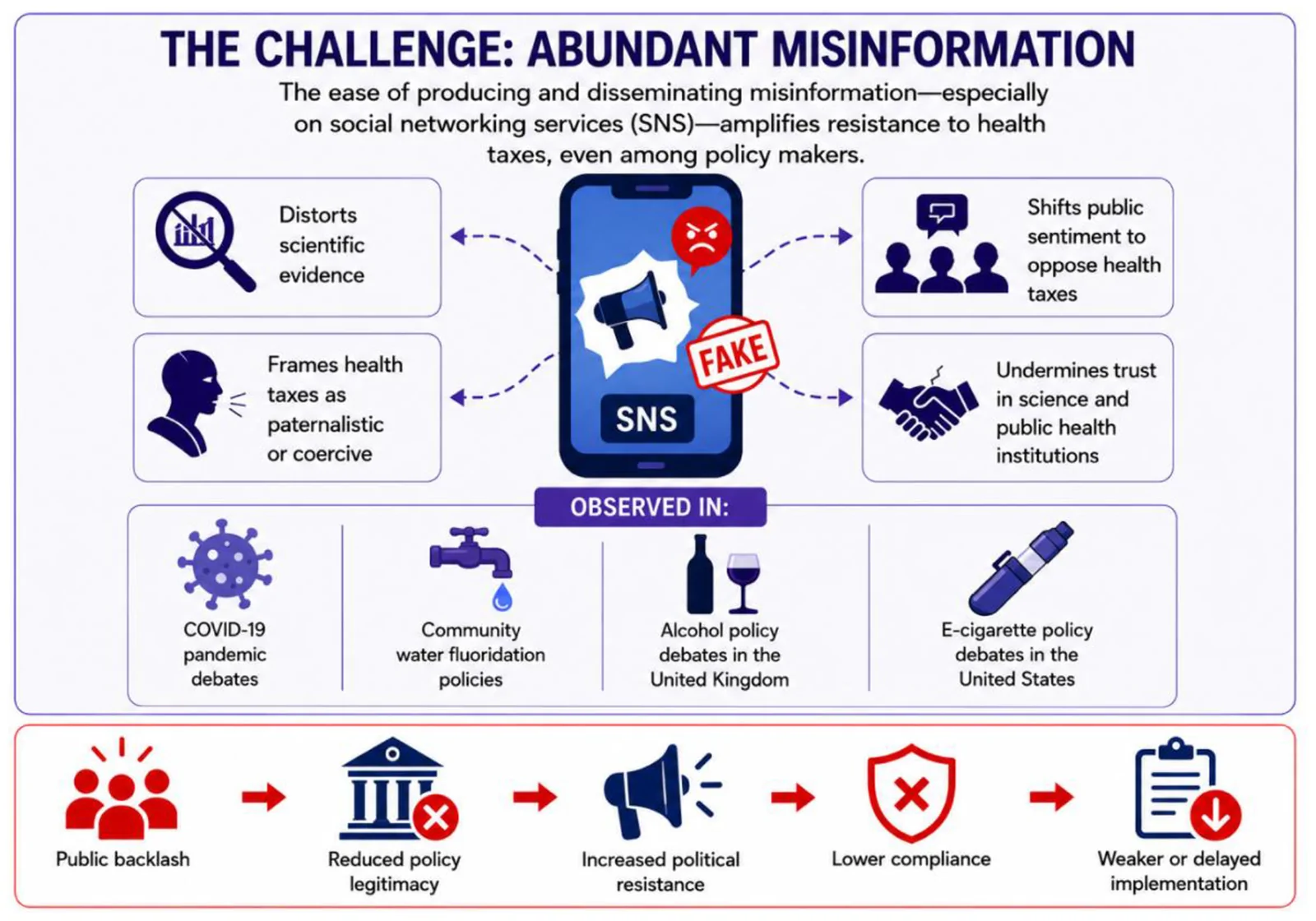
The effect of abundant misinformation on the debate on health taxes. Open AI's ChatGPT free version was used to generate this figure.

Misinformation affects not only public opinion, but also policy acceptance and compliance (9, 10). When individuals are exposed to conflicting or misleading information about the effectiveness or the purpose of health taxes, they may perceive such policies as unjustified or coercive (25, 26). This can lead to public backlash, reducing policy legitimacy and increasing political resistance (20, 27). A systematic review and meta-analysis of 40 studies showed that beliefs about the effectiveness and appropriateness of SSB taxes was one of the major determinants of public and political acceptability of the SSB taxes (30). This is highly relevant to misinformation because directly targeting the public's beliefs about health taxes could influence tax acceptance. In addition, a discrete-choice experiment from the United Kingdom showed that public acceptance of minimum unit pricing (MUP) of alcohol, a policy aimed to reduce alcohol consumption, increased upon informing the participants about the expected health outcomes of this policy compared to when they were not informed about these health outcomes (31). Furthermore, misinformation can exacerbate the perceived tension between public health goals and personal freedom, framing health taxes as governmental overreach rather than evidence-based interventions (6, 19). A detailed policy case study from Scotland involving parliament documents analysis and 36 interviews with stakeholders showed that the industry primarily framed MUP of alcohol as a problem of social disorder caused by a minority of young or problematic drinkers, thereby promoting targeted rather than population-wide interventions, however, public-health advocates, by contrast, framed alcohol as a population-level health problem requiring population-wide action (32). The study concluded that deliberate changes in policy framing helped prioritize public health considerations in the policy debate (32).

Winning the public sentiment battle and gaining public support on health taxes should not be underestimated as public backlashes have led to reversing health policies in the past (20). For example, there was a quick reversal of a smoking ban targeting the young generation in New Zealand in early 2024 after a year of implementing it in 2022 (20, 33). Similar quick scrapping of sugar taxation policies was observed in the middle east and north Africa because of the cultural perception of SSBs (SSBs are perceived as essential treats despite their health risk) in the region among other reasons (20).

## What should be done?

Addressing these challenges requires a comprehensive approach (Figure 2). First, transparent communication of scientific evidence on digital and social media platforms using contemporary presentation formats (short format videos (reels), long format videos, podcasts and documentaries) is essential (34). Public health authorities and advocates must clearly articulate the rationale, effectiveness, and the intended benefits of health taxes, using easily understood language and credible sources, while publicly acknowledging the knowledge gaps and uncertainties of the available scientific evidence (26, 34). Communication strategies that emphasize the strength of expert consensus and actively debunking misinformation have been shown to reduce misperceptions and enhance trust in science (35, 36). Second, public health authorities and advocates should show openness to de-implementation of the health taxes if credible evidence emerged on its ineffectiveness in reducing the burden of NCDs in certain settings such as during sudden economic crunches or in certain regions (20, 26). Third, researchers and academics should provide scientific evidence with clear causal answers in different settings and regions to address the research gaps, and adopt the iterative learning process with humility (26). Fourth, engaging communities in the policy-making process through citizens deliberations can enhance trust and ensure that interventions are culturally and socially acceptable (37). Fifth, a gradual phased implementation of health taxes, rather than a one-time implementation, could allow reduction of the political risk by smoothing the economic shock on consumers, and giving time to show the benefits of earmarking the tax revenues for health services, and thus refuting regressivity claims, and at the same time build constituencies for subsequent tax increments (27). Sixth, the effectiveness of health taxes may depend on the broader policy environment. Standalone fiscal measures are more likely to succeed when integrated into comprehensive public health strategies that include complementary measures such as subsidies for healthy alternatives, targeted health promotion programs, direct reinvestment of tax revenues into underserved communities, combining tobacco and SSB taxes with front-of-package labeling and restrictions on marketing to children (11, 13, 16, 20). This can address the potential financial burdens on low socioecnomic groups and produce synergistic effects, and help aligning public health objectives with social justice considerations (11, 13, 16). Such integrated approaches recognize that health behaviors are shaped by complex systems rather than isolated factors (18).

**Figure 2:**
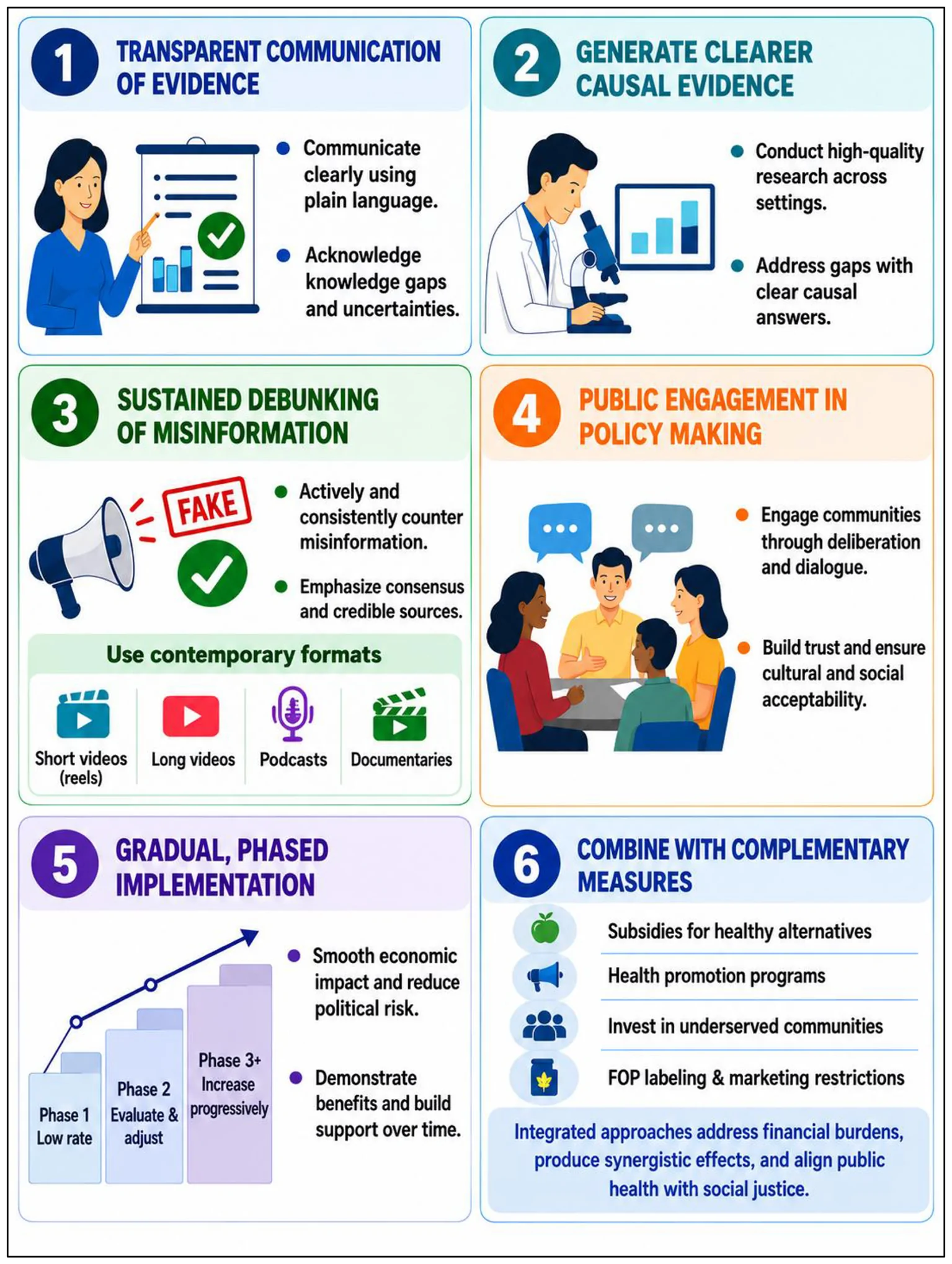
The suggested way forward to tackle the effect of misinformation on the implementation of health taxes. FOP labeling, front-of-package labeling; Open AI's ChatGPT free version was used to generate this figure.

## Conclusion

Health taxes represent a powerful tool for improving population health, particularly in the prevention of non-communicable diseases. While concerns about individual freedom of choice are valid and warrant careful consideration, these policies do not eliminate autonomy but rather seek to rebalance the environment in which consumption choices are made. The challenges of implementing health taxes in an era of abundant misinformation are compounded due to the ease of disseminating misinformation at a scale. Misinformation can amplify the resistance to health taxes by shifting the public sentiment to oppose health taxes through distorting scientific evidence, framing health taxes as paternalistic or coercive, and undermining trust in science and public health institutions. To address these challenges, transparent communication, sustained debunking of misinformation, public engagement in policy making, gradual phased implementation of health taxes, and combining health taxes with complementary measures such as providing subsidies for healthy alternatives among other tools might be useful to build trust, win public support for health taxes and promote informed decision-making.
